# Failure of subcutaneous lipectomy to combat metabolic dysregulations in ovariectomy-induced obesity in young female rats

**DOI:** 10.1007/s42000-022-00371-0

**Published:** 2022-04-29

**Authors:** Bataa El-Kafoury, Fatma Mohamed, Nehal Bahgat, Abeer Abd El Samad, Mona Shawky, Enas A. Abdel-Hady

**Affiliations:** 1 Physiology Department, Faculty of Medicine, Ain Shams University, Cairo, Egypt; 2 Histology Department, Faculty of Medicine, Ain Shams University, Cairo, Egypt

**Keywords:** Lipectomy, Obesity, Ovariectomy, Subcutaneous adipose tissue, Visceral adipose tissue

## Abstract

**Purpose:**

The deleterious effect of visceral adipose tissue accumulation is well known. However, the recent trend in liposuction is mal-directed toward easily accessible subcutaneous fat for the purpose of body shaping. The aim of the present study is to probe the metabolic effects of subcutaneous abdominal adipose tissue lipectomy in ovariectomized obese rats as well as the role of adipokines in these changes.

**Methods:**

The study was conducted on young female rats randomized into two main groups according to the duration of the experiment, namely, 5-week and 10-week. Both groups were subdivided as follows: sham-operated, ovariectomized, and ovariectomized lipectomized rat groups. The rats underwent measurement of body weight (BW) and determination of body mass index (BMI). Fasting blood glucose, lipid profile, liver function, plasma malondialdehyde, leptin, and adiponectin were estimated, and the content of both blood and hepatic tissue of reduced glutathione was assessed. In addition, histological study of the liver, aorta, and perirenal fat of all rat groups was performed.

**Results:**

Ovariectomy-induced obesity is marked by a significant increase in BW and BMI. Following subcutaneous lipectomy, the rats exhibited significant weight gain accompanied by fasting hyperglycemia, dyslipidemia, deterioration of synthetic function of the liver, and disturbed oxidant/antioxidant status. Histological examination revealed fatty infiltration of aortic and hepatic tissues.

**Conclusion:**

Despite the immediate positive effect of subcutaneous lipectomy for weight loss and/or body shaping, multiple delayed hazards follow the procedure, which should be carefully considered.

## Introduction

White fat depots have been recognized to be not merely inert lumps: besides their well-known energy storing function, these depots are actually sites in the body made up of endocrine tissue that secretes physiologically active molecules, i.e., adipokines with their variable metabolic effects [1]. Adipocyte-derived factors include leptin, which serves as a peripheral signal directing the central nervous system to adjust food intake and energy expenditure in accordance with the amount of energy reserve [2]. Adiponectin, the most abundant adipokine, runs counter to leptin by exhibiting a strong negative correlation between its plasma concentration and body mass index; it is also known for its peripheral metabolic antidiabetic and anti-atherogenic effects [3]. Resistin, omentin, and retinol binding protein-4 have all been shown to play significant roles in obesity-induced insulin resistance [4, 5]. Apelin is a peptide found in both the stromal-vascular cells and in differentiated adipocytes and which stimulates angiogenesis [6].

White adipose tissue (WAT) is distributed throughout the body in two forms, subcutaneous adipose tissue (SAT) and visceral adipose tissue (VAT). SAT stores >80% of total body fat and is most commonly present in abdominal, gluteal, and femoral depots [7]. SAT is independently correlated with metabolic complications of obesity and has been shown to respond better to the antilipolytic effects of insulin and other hormones [8]. On the other hand, VAT, which is associated with internal organs, is reported to represent 10–20% of total body fat in men and 5–10% in women [9]. The deleterious effect of VAT is attributed to its greater expression of proinflammatory cytokines compared to SAT [10]. Studies in rodents have demonstrated improved glucose tolerance, reversal of insulin resistance, and reduced adipo/cytokine levels following the removal of visceral or intra-abdominal adipose tissue [11]. The studies reported improvement in insulin sensitivity and glucose tolerance with SAT transplantation into the visceral cavity [12], but not with VAT transplantation [13]. These data along with the various characteristics mentioned above contrasting VAT with SAT point to the importance of SAT.

For over four decades, due to culturally defined beauty standards and the increasing rate of obesity, surgical removal of adipose tissue has been ever more widely employed, with, perhaps not surprisingly, liposuction being the most popular cosmetic procedure in the world. However, studies conducted to evaluate the efficacy of lipectomy have been inconclusive and its effect on metabolism remains unclear. The problem is that today liposuction surgery and/or low level laser therapy for body contouring and reduction of cellulite are mal-directed toward SAT, especially in the abdominal region due to its easier access as compared to that of VAT [14]. This trend necessitates extensive study of the consequences of lipectomy, especially as obesity rates continue to rise in young women. National surveys from a range of countries report larger increases in weight in young women (aged 18−35) in recent years compared to those seen in older women [15, 16].

In the present study, we selected surgical bilateral ovariectomy as an experimental model of obesity to investigate the metabolic responses toward subcutaneous lipectomy and to probe how leptin and adiponectin could contribute to these metabolic changes in young female rats.

## Materials and methods

### Experimental protocol

All experimental and surgical procedures were approved by the Research Ethics Committee (REC), Faculty of Medicine, Ain Shams University, Cairo, Egypt (protocol number FMASU 1162/2012). The study was performed on 72 young (3 months) female Wistar rats initially weighing 180–220 g, which were randomly allocated into two main groups, as follows:

A) 5-week group to study the early effects of lipectomy. It was further subdivided as follows:

1. Sham-operated: Rats were subjected to all steps of the ovariectomy operation without removal of the ovaries. They were sacrificed 5 weeks later and served as the control group (n = 10).
2. Ovariectomized (OVX): Rats were bilaterally ovariectomized, then were sacrificed 5 weeks later (n = 13).
3. Ovariectomized and lipectomized (OVXL): Rats were bilaterally ovariectomized, and then subjected to lipectomy 4 weeks later. They were sacrificed 1 week following the lipectomy operation (n = 13).

B) 10-week group to study the late effects of lipectomy. It was further subdivided as follows

1. Sham-operated: Rats were subjected to all steps of the ovariectomy operation without removal of the ovaries. They were sacrificed 10 weeks later and served as the control group (n = 10).
2. Ovariectomized (OVX): Rats were bilaterally ovariectomized, then were sacrificed 10 weeks later (n = 13).
3. Ovariectomized and lipectomized (OVXL): Rats were bilaterally ovariectomized, and then subjected to lipectomy 4 weeks later. They were sacrificed 6 weeks following the lipectomy operation (n = 13). Bilateral surgical ovariectomy (as a model of human obesity) and subcutaneous belt lipectomy were performed according to the steps described in our previous research [17].

Throughout the study, body weight (BW) was measured and body mass index (BMI) was calculated. On the day of sacrifice, fasting blood glucose (FBG) was estimated via a tail blood sample using the OneTouch apparatus and Uni-Check blood glucose test strips. The rats were then anesthetized by intraperitoneal injection of thiopental sodium (Sandoz, Austria) at a dose of 50 mg/kg. A midline abdominal incision was made; the abdominal aorta was exposed and cannulated for blood sampling. The liver was then removed and weighed and its ratio to BW was calculated. The right lobe was washed in ice-cold saline, blotted by filter paper, wrapped in parafilm, and stored at −80°C for later determination of reduced glutathione level. Perirenal fat pads were dissected and weighed, and the ratio of their weight to BW was calculated.

### Biochemical studies

Reduced glutathione (GSH), plasma proteins, liver enzymes, lipid profile, malondialdehyde (MDA), and hormonal levels were measured with the enzymatic colorimetric technique using commercially available kits according to the manufacturer’s instructions. The right lobe of the liver was homogenized in 5 ml cold buffer (50 mM potassium phosphate, pH 7.5, and 1 mM EDTA) per gram tissue using tissue homogenizer (IKA-WERK, Ultra-Turrax, Germany), and then centrifuged at 4000 rpm for 15 min. The supernatant was used for measurement of GSH in hepatic tissue with the same kit used for blood analysis.

### Histological study

The perirenal fat pads, the left lobe of the liver and, a segment of the abdominal aorta proximal to the canulated part were fixed in 10% formalin solution immediately after removal. The specimens were dehydrated in ascending grades of alcohol, cleared in xylene, and embedded in paraffin. Serial sections of 5 mm thickness were cut and stained with hematoxylin and eosin (H&E) for evaluation of the histological changes. The average cell size of adipocytes was measured using image analyzer (Leica Q win V.3 program installed on a computer connected to Leica DM2500 microscope Wetzlar, Germany). The area of individual fat cell (μm^2^) was measured in ten fields of two serial sections of VAT of seven rats of each group using ×10 power lens, and a mean value was calculated for each field. Then, a mean value was calculated for all fields of each rat.

### Statistical analysis

Results were expressed as median (minimal value; maximal value). Percentage (%) changes of BW and BMI were calculated relative to their initial values. Statistical significance between groups was determined by the Kruskal-Wallis test and Mann-Whitney U test. The Wilcoxon signed-rank test was used to detect the statistical significance of paired variables within the same group**.** Correlation between variables was evaluated using Spearman’s rho correlation coefficient. Statistical analysis was performed by using Statistical Package for the Social Sciences (SPSS) software version 20.0 (SPSS Inc., Chicago, Illinois, USA), and a probability of *p* < 0.05 was considered statistically significant.

## Results

### Body weight and body mass index

**Fig. 1:**
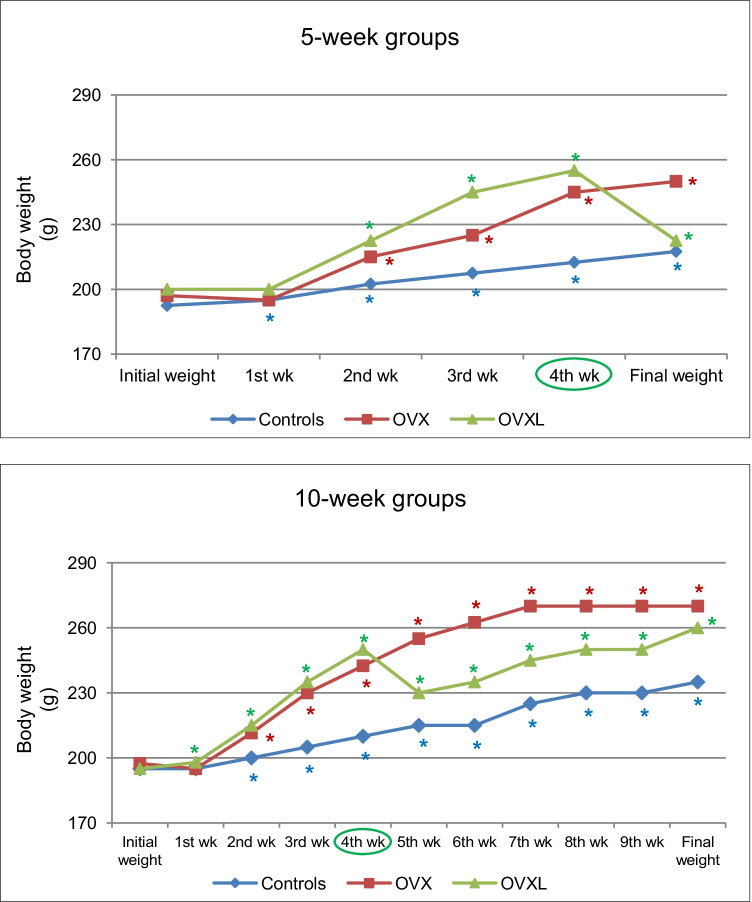
Body weight changes in sham-operated (controls), ovariectomized (OVX), and ovariectomized lipectomized (OVXL) rats. Graphs representing the weekly changes in body weight as median values. *: Significance of differences from initial body weight of the same group was calculated with the Wilcoxon signed-rank test at *p* < 0.05 The time of lipectomy surgery is indicated by a green circle

The weekly measured BW revealed a steady increase in all rat groups except for the abrupt decrease 1 week after subcutaneous lipectomy in both 5-week and 10-week OVXL rats. Overall, the OVX rats showed a higher slope of weight gain over time. Surprisingly, the abrupt decrease in BW observed 1 week after lipectomy in the 10-week OVXL rats was followed by a steady increase in BW thereafter to become unrecognizably different from the 10-week only OVX rats (Fig. 1). The percentage increase in BW and BMI was significantly higher in both 5-week and 10-week OVX rats compared to their corresponding sham-operated (control) groups (*p* < 0.001 for all). The percentage increase was also higher in the 10-week OVX rats compared to the 5-week OVX rats (*p* < 0.001). Upon lipectomy, these ratios were significantly reduced in the 5-week OVXL rats compared to the 5-week only OVX group (*p* < 0.001). However, 6 weeks after lipectomy, no changes were observed in these ratios compared to the 10-week only OVX rats, although it was significantly higher compared to the control values and the 5-week OVXL group (*p* < 0.001 for all) (Table 1).

**Table 1:** Body weight (BW), body mass index (BMI), perirenal fat, and liver weight in the studied groups

	5-week			10-week		
	Control	OVX	OVXL	Control	OVX	OVXL
Final BW (g)	217.5 (200, 230)	250 (220, 270)^a^	222.5 (190, 235)^b^	235 (190, 250)	270 (250, 305)^a*^	260 (220, 350)^a†^
Δ BW (%)	11.56 (5.00, 18.92)	22.73 (18.42, 33.33)^a^	8.72 (−2.27, 17.50)^b^	19.05 (10.00, 32.35)	42.11 (33.33, 47.78)^a*^	34.52 (21.05, 59.09)^a†^
Final BMI (g/cm^2^)	0.52 (0.49, 0.52)	0.57 (0.52, 0.60)^a^	0.50 (0.43, 0.55)^b^	0.53 (0.51, 0.56)	0.63 (0.57, 0.64)^a*^	0.59 (0.52, 0.67)^a†^
Δ BMI (%)	1.98 (1.96, 2.08)	16.67 (14.58, 21.28)^a^	0.00 (−10.42, 4.17)^b^	7.69 (3.77, 8.33)	27.34 (21.15, 31.25)^a*^	21.15 (12.77, 37.50)^a†^
Perirenal fat (g)	3.30 (1.70, 5.80)	4.50 (3.60, 6.80)^a^	4.35 (2.30, 6.00)	1.60 (1.20, 2.10)	7.65 (5.40, 9.20)^a*^	5.80 (3.00, 13.10)^a†^
Perirenal fat/BW (%)	1.46 (0.74, 2.90)	1.77 (1.46, 2.56)	2.04 (1.05, 2.73)	0.68 (0.53, 0.88)	2.78 (2.16 , 3.07)^a*^	2.35 (1.21, 4.23)^a^
Liver weight (g)	4.80 (4.10, 5.40)	6.20 (5.10, 7.30)^a^	6.75 (4.30, 7.90)^a^	4.70 (3.60, 7.10)	5.40 (4.50, 6.40)^*^	4.90 (3.70 ,7.30)^†^
Liver weight/BW (%)	2.23 (1.86, 2.45)	2.67 (2.08, 2.84)^a^	2.97 (2.00, 3.63)^ab^	2.04 (1.67, 3.02)	1.88 (1.67, 2.22)^*^	1.88 (1.67, 2.09)^a†^

Values are presented as median (min, max), n = 10 for controls; n = 13 for OVX and OVXL

Significance of differences between groups was calculated with the Mann-Whitney U test at *p* < 0.05: a, vs. corresponding controls; b, vs. corresponding OVX rats; *, 5-week OVX vs. 10-week OVX; †, 5-week OVXL vs. 10-week OVXL

*OVX*, ovariectomized rats; *OVXL*, ovariectomized lipectomized rats

### Perirenal fat mass

Only absolute perirenal fat mass was significantly increased in the 5-week OVX rats compared to sham-operated control group (*p* = 0.007). However, the 10-week OVX rats showed significant increase in both absolute and relative perirenal fat mass compared to controls as well as to the 5-week OVX rats (*p* < 0.001 for all). Upon lipectomy, the 10-week OVX rats showed an increase in both values when compared to the sham-operated rats (*p* < 0.001), but there was no difference when compared to the 10-week only OVX group. In addition, absolute perirenal fat was significantly higher in the 10-week OVXL than the 5-week OVXL rats (*p* = 0.031) (Table 1).

### Liver weight

Both absolute and relative liver weights were significantly increased in the 5-week OVX rats compared to their matched controls (*p* < 0.001 and *p* = 0.017, respectively). Meanwhile, the 10-week OVX rats showed significant reduction in both values compared to the 5-week OVX rats (*p* = 0.011 and *p* < 0.001, respectively). Following lipectomy, the absolute and relative liver weights were also significantly higher in the 5-week OVXL rats than in controls (*p* = 0.001 for both), and the relative weight was higher than in the corresponding OVX rats (*p* = 0.012). Notably, both values were significantly reduced in the 10-week OVXL rats compared to the 5-week OVXL group (*p* = 0.002 and *p* < 0.001, respectively) (Table 1).

### Blood glucose and lipid profile

FBG level was significantly increased in the 5-week OVX rats compared to their matched controls (*p* = 0.014), while in the 10-week OVX rats, the values of FBG were similar to those of the corresponding sham-operated group. Following lipectomy, FBG was significantly increased in both the 5-week and the 10-week rats compared to their corresponding only OVX rats (*p* = 0.002 and *p* = 0.007, respectively) as well as the sham-operated groups (*p* < 0.001 and *p* = 0.002, respectively) (Table 2).

**Table 2:** Biochemical parameters of the studied groups

	5-week			10-week		
	Control	OVX	OVXL	Control	OVX	OVXL
FBG (mg/dl)	70.00 (62.00, 76.00)	85.00 (61.00, 96.00)^a^	102.00 (80.00, 144.00)^ab^	70.00 (63.00, 78.00)	67.00 (53.00, 75.00)^*^	81.00 (61.00, 135.00)^ab†^
TG (mg/dl)	39.74 (25.12, 56.81)	48.86 (25.00, 82.13)	63.20 (32.85, 104.76)^a^	33.94 (21.85, 49.58)	42.02 (33.61, 52.96)^a^	39.50 (25.21, 62.18)^†^
TC (mg/dl)	59.44 (55.10, 85.19)	78.70 (52.78, 87.96)^a^	70.91 (62.04, 92.59)^ab^	71.95 (51.06, 92.91)	76.60 (68.10, 96.32)	88.65 (73.76, 121.28)^ab†^
HDL-C (mg/dl)	39.88 (30.41, 48.81)	43.03 (33.33, 49.72)	28.66 (20.83, 34.43)^ab^	45.71 (23.97, 69.78)	49.50 (41.39, 64.91)^*^	49.50 (40.32, 66.02)^†^
LDL-C (mg/dl)	13.68 (10.66, 25)	21.64 (14.13, 34.30)^a^	31.35 (21.03, 53.15)^ab^	18.05 (13.38, 27.22)	20.06 (13.76, 28.43)	31.92 (20.32, 47.67)^ab^
AI	0.58 (0.39, 0.81)	0.84 (0.58, 1.00)^a^	1.59 (1.22, 1.98)^ab^	0.57 (0.33, 0.86)	0.54 (0.43, 0.78)^*^	0.79 (0.57, 1.14)^ab†^
Total proteins (g/dl)	6.53 (6.34, 7.45)	6.24 (5.55, 6.91)^a^	5.22 (4.88, 5.65)^ab^	5.80 (5.44, 7.09)	5.94 (5.59, 6.21)^*^	6.72 (5.86, 7.26)^ab†^
Albumin (g/dl)	3.91 (3.01, 4.62)	3.62 (3.01, 4.82)	2.26 (2.02, 3.33)^ab^	3.56 (3.21, 4.18)	2.99 (2.46, 3.71)^a*^	2.36 (2.10, 2.51)^ab^
ALT (IU/l)	28.92 (24.11, 30.80)	34.82 (31.25, 44.64)^a^	21.43 (16.07, 27.68)^ab^	26.78 (21.88, 33.93)	35.30 (28.57, 42.86)^a^	30.00 (22.32, 41.96)^†^
AST (IU/l)	52.89 (44.23, 78.85)	75 (63.46, 88.46)^a^	55.19 (48.08, 69.23)^b^	54.81 (49.62, 63.46)	51.92 (48.08, 62.50)^*^	56.73 (42.31, 72.12)
Plasma MDA (nmol/ml)	32.50 (27.00, 39.00)	105.00 (64.00, 136.00)^a^	173.50 (128.00, 229.00)^ab^	42.00 (26.00, 58.00)	122.50 (102.00, 161.00)^a*^	194.00 (166.00, 221.00)^ab^
Blood GSH (mg/dl)	27.53 (25.14, 33.60)	26.26 (19.86, 30.06)	16.74 (10.00, 20.40)^ab^	22.40 (19.46, 25.53)	24.66 (20.53, 27.13)	23.00 (19.46, 25.20)^†^
Hepatic GSH (mg/g)	34.21 (29.74, 45.23)	24.39 (22.08, 27.95)^a^	15.16 (10.38, 17.23)^ab^	31.71 (25.19, 47.59)	23.60 (20.67, 28.96)^a^	12.75 (10.55, 18.61)^ab^
Leptin (pg/ml)	50.75 (31.60, 91.39)	179.66 (111.03, 330.04)^a^	89.35 (12.81, 238.51)^b^	37.28 (32.95, 59.47)	142.55 (115.43, 218.77)^a^	29.98 (8.15, 135.62)^b^
Adiponectin (ng/ml)	20.12 (13.18, 27.90)	17.02 (13.17, 23.47)	19.54 (15.09, 21.31)	15.88 (13.69, 17.25)	15.98 (12.70, 17.40)	16.34 (11.53, 18.48)^†^

Values are presented as median (min, max), n = 10 for controls; n = 13 for OVX and OVXL

Significance of differences between groups was calculated with the Mann-Whitney U test at *p* < 0.05: a, vs. corresponding controls; b, vs. corresponding OVX rats; *, 5-week OVX vs. 10-week OVX; †, 5-week OVXL vs. 10-week OVXL

*OVX*, ovariectomized rats; *OVXL*, ovariectomized lipectomized rats; *FBG*, fasting blood glucose; *TG*, triglycerides; *TC*, total cholesterol; *HDL-C*, high-density lipoprotein-cholesterol; *LDL-C*, low-density lipoprotein-cholesterol; *AI*, atherogenic index; *ALT*, alanine aminotransferase; *AST*, aspartate aminotransferase; *MDA*, malondialdehyde; *GSH*, reduced glutathione

Plasma total cholesterol (TC), LDL-C, and the calculated atherogenic index (AI) were all significantly increased in the 5-week OVX rats compared to the corresponding sham-operated group (*p* = 0.005, *p* = 0.006, and *p* = 0.007, respectively). With prolonged duration, the 10-week OVX rats showed an increase in plasma HDL-C associated with a decrease in AI compared to the 5-week OVX group (*p* = 0.003 and *p* = 0.002, respectively). Following lipectomy, the 5-week OVXL rats showed a significant rise in plasma LDL-C and AI accompanied by a significant decrease in HDL-C as compared to both controls (*p* < 0.001 for all) and OVX rats (*p* = 0.014, *p* < 0.001, and *p* < 0.001, respectively), while plasma triglycerides (TG) and TC were higher only in comparison to control rats (*p* = 0.001 and *p* = 0.006, respectively). Similarly, the 10-week OVXL rats showed significant increases in levels of TC, LDL-C, and AI compared to controls (*p* = 0.006, *p* < 0.001, and *p* = 0.001, respectively) and to the OVX rats (*p* = 0.039, *p* < 0.001, and *p* < 0.001, respectively) (Table 2).

### Liver functions

Plasma total proteins were significantly decreased in the 5-week OVX rats compared to the corresponding sham controls (*p* = 0.026). After 1 week of subcutaneous lipectomy in the 5-week OVX rats, there was significant reduction in plasma total proteins compared to the only OVX rats and also to the sham controls (*p* < 0.001 for both). Meanwhile, in the 10-week OVXL rats, it was significantly increased compared to the corresponding OVX rats and controls (*p* = 0.001 and *p* = 0.006, respectively), as well as in the 5-week OVXL rats (*p* < 0.001) (Table 2).

Plasma albumin showed no change in the 5-week OVX rats compared to the matched controls; however, it was significantly decreased in the 10-week OVX rats compared to their corresponding controls and to the 5-week OVX rats (*p* = 0.002 and *p* = 0.004, respectively). Upon lipectomy, albumin level was significantly reduced in both the 5-week and the 10-week OVXL rats compared to their corresponding only OVX and sham-operated groups (*p* < 0.001 for all) (Table 2).

The plasma level of alanine aminotransferase (ALT) and aspartate aminotransferase (AST) was significantly elevated in the 5-week OVX rats compared to the corresponding sham controls (*p* < 0.001 and *p* = 0.001, respectively). In the 10-week OVX rats, ALT was still significantly elevated compared to controls (*p* < 0.001), but no difference was detected when compared to the 5-week OVX rats. AST was, however, significantly decreased in the 10-week group compared to the 5-week OVX group (*p* < 0.001). One-week OVXL rats had lower levels of both ALT and AST compared to the OVX rats (*p* < 0.001 for both), while compared to controls, only ALT was significantly reduced (*p* < 0.001). Six weeks later, ALT was significantly higher only in comparison to the 5-week OVXL group (*p* = 0.002), with no statistical differences in AST level compared to the corresponding controls, the OVX group, and even the 5-week rats (Table 2).

### Oxidant/antioxidant status

Plasma MDA was significantly increased in both the 5-week and the 10-week OVX rats compared to their corresponding control groups (*p* < 0.001 for both), being significantly higher in the 10-week OVX compared to the 5-week OVX rats (*p* = 0.002). Moreover, after lipectomy, it was significantly higher in both the 5-week and the 10-week OVXL rats compared to their corresponding only OVX rats as well as to the sham-operated groups (*p*< 0.001 for all) (Table 2).

No significant difference was detected in the level of blood GSH among the OVX groups (5-week and 10-week) or between them and their matched controls. However, hepatic tissue levels of GSH were significantly reduced in both the 5-week and the 10-week OVX groups compared to their controls (*p* < 0.001). Following lipectomy, both blood and hepatic GSH were significantly decreased in the 5-week OVXL rats compared to their corresponding values in the only OVX rats as well as the sham-operated controls (*p* < 0.001 for all). Meanwhile, only hepatic GSH was significantly reduced in the 10-week OVXL group compared to both only OVX rats and sham-operated controls (*p* < 0.001) (Table 2).

### Hormonal levels

Plasma leptin level was significantly increased in both the 5-week and the 10-week OVX rats compared to their corresponding sham-operated control groups (*p* = 0.002). At the same time, there was no statistical difference in its level among the two OVX groups. Following lipectomy, leptin level was significantly reduced in the 5-week OVXL as well as in the 10-week OVXL rats compared to the matched only OVX rats (*p* = 0.006 and *p* = 0.002, respectively). On the other hand, adiponectin levels were statistically indifferent among all the studied rat groups (Table 2).

### Histological study

**Fig. 2:**
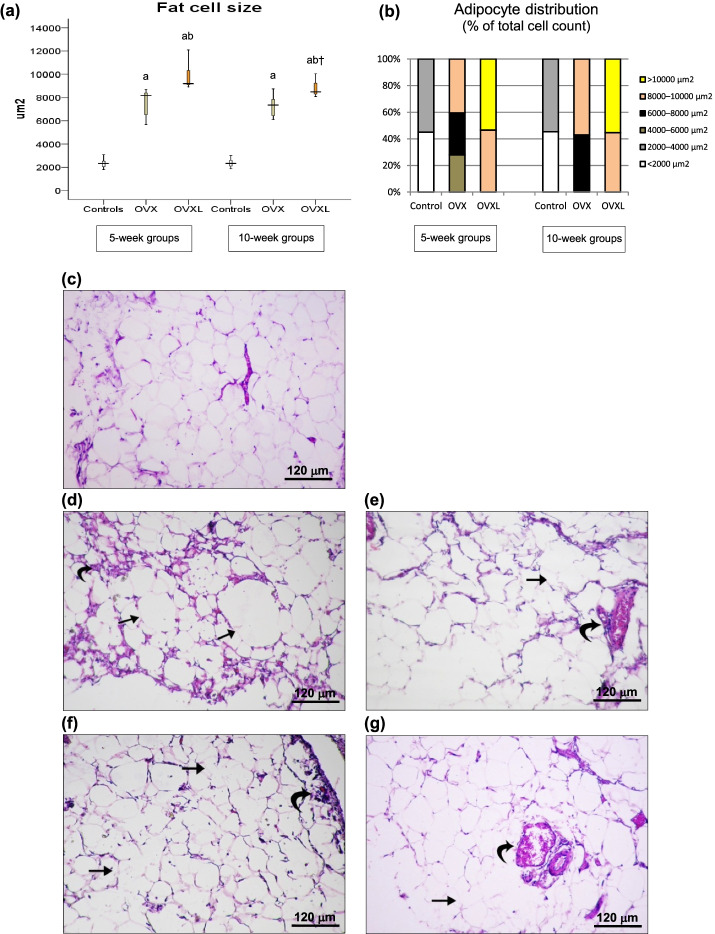
Visceral adipose tissue in sham-operated (controls), ovariectomized (OVX), and ovariectomized lipectomized (OVXL) rats. (**a**) Fat cell size presented as median (min, max), n = 7/group, significance of differences was calculated with the Mann-Whitney U test at *p* < 0.05: a, vs. corresponding controls; b, vs. corresponding OVX rats; †, 5-week OVXL vs. 10-week OVXL; (**b**) Adipocytes distribution ranges; (**c**) Light photomicrographs of sham-operated controls showing normal appearance of white fat cells with thin rim of cytoplasm surrounding a large vacuole of dissolved lipid and flattened peripherally situated nuclei; (**d**) 5-week OVX rats showing larger fat cells (thin arrow) with irregular membrane and inflammatory cellular infiltration around blood vessels (curved arrow); (**e**) 10-week OVX rats showing destruction of membranes between fat cells (thin arrow) with appearance of larger irregular fat cells and inflammatory cellular infiltration around large congested blood vessels (curved arrow); (**f**) 1-week lipectomized rats showing distorted membranes between most of adjacent fat cells (thin arrow) giving larger irregular fat cells with inflammatory cellular infiltration between fat cells and around blood vessels (curved arrow); (**g**) 6-week lipectomized rats showing distorted membranes between most of adjacent fat cells (thin arrow) with large irregular fat cells and increasing congested vasculature between fat cells with inflammatory cellular infiltration (curved arrow)

As shown in Fig. 2a,b, perirenal fat cell size was significantly increased in both the 5-week and the 10-week OVX rats compared to the corresponding sham-operated control groups (*p* = 0.002 for both), with no difference between the two OVX groups. It was further increased in the 5-week and the 10-week OVXL rats compared to both the only OVX rats (*p* = 0.002, *p* = 0.018, respectively) and the sham controls (*p* = 0.002 for both).

Histological examination of VAT of control rats showed the normal appearance of white fat cells with a thin rim of cytoplasm surrounding a large vacuole of dissolved lipid and peripherally situated, flattened nuclei (Fig. 2c). Five weeks after ovariectomy, large fat cells with irregular membrane appeared compared to controls. In addition, perivascular inflammatory cell infiltration was observed (Fig. 2d), whereas after 10 weeks, the membranes between the fat cells were destroyed, resulting in larger and irregular fat cells. Inflammatory cell infiltration could still be observed around large congested blood vessels (Fig. 2e). One week following lipectomy, distortion of membranes occurred between most of the adjacent fat cells, giving rise to larger irregular cells, along with a cellular inflammatory infiltrate between fat cells and around the blood vessels (Fig. 2f). After 6 weeks, the condition worsened, with further distortion of the intercellular membranes and the appearance of more large irregular fat cells with increased vascular congestion and infiltration of inflammatory cells (Fig. 2g).

**Fig. 3:**
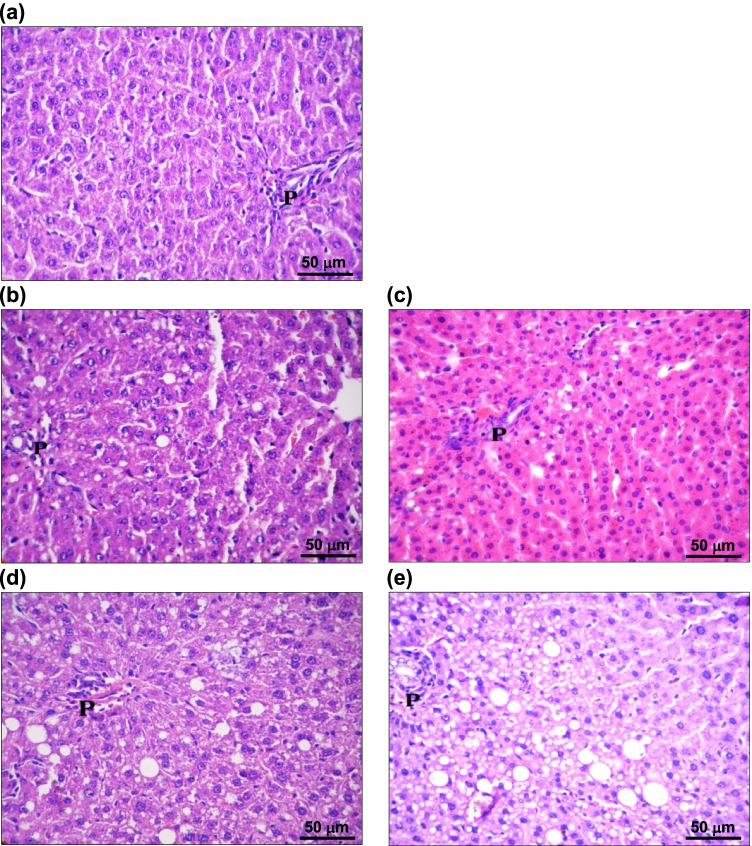
Light photomicrographs of rats’ liver sections. (**a**) Sham-operated rats showing hepatocytes with central round vesicular nuclei and acidophilic cytoplasm nearby portal tract (P); (**b**) 5-week ovariectomized rats showing swollen hepatocytes, some with small and others with large vacuoles (dissolved fat droplets) around a portal tract (P) with congested blood sinusoids; (**c**) 10-week ovariectomized rats showing vacuolated hepatocytes with dissolved fat droplets around a portal tract (P), which shows congested blood vessels; (**d**) 1-week lipectomized rats showing increased vacuolation of hepatocytes (ballooning of hepatocytes with dissolved fat droplets) and inflammatory cellular infiltration in portal tract (P) with decreased size of blood sinusoids; (**e**) 6-week lipectomized rats showing increased number of swollen variable sized hepatocytes with dissolved fat droplets and flattened peripherally situated nuclei around a portal tract (P)

Liver sections from sham-operated rats showed the normal architecture with a central vein surrounded by branching cords of hepatocytes and portal tracts at the corners of the hepatic lobule. The hepatocytes have central, round vesicular nuclei and acidophilic cytoplasm (Fig. 3a). The 5-week OVX rats showed vacuolation of some hepatocytes in the branching cords between portal tracts (Fig. 3b). Ten weeks following ovariectomy, vacuolation was found to extend to the central veins, accompanied by an increase in inflammatory cellular infiltration in the portal tracts (Fig. 3c). One week after lipectomy, there was an increase in vacuolation and ballooning of hepatocytes around the portal tracts and their extension to central veins, accompanied by increased inflammatory cellular infiltration (Fig. 3d). In the 10-week OVXL group, there were still an increased number of swollen hepatocytes of variable size and flattened peripheral nuclei, as well as signs of fatty degeneration around the portal tracts (Fig. 3e).

**Fig. 4:**
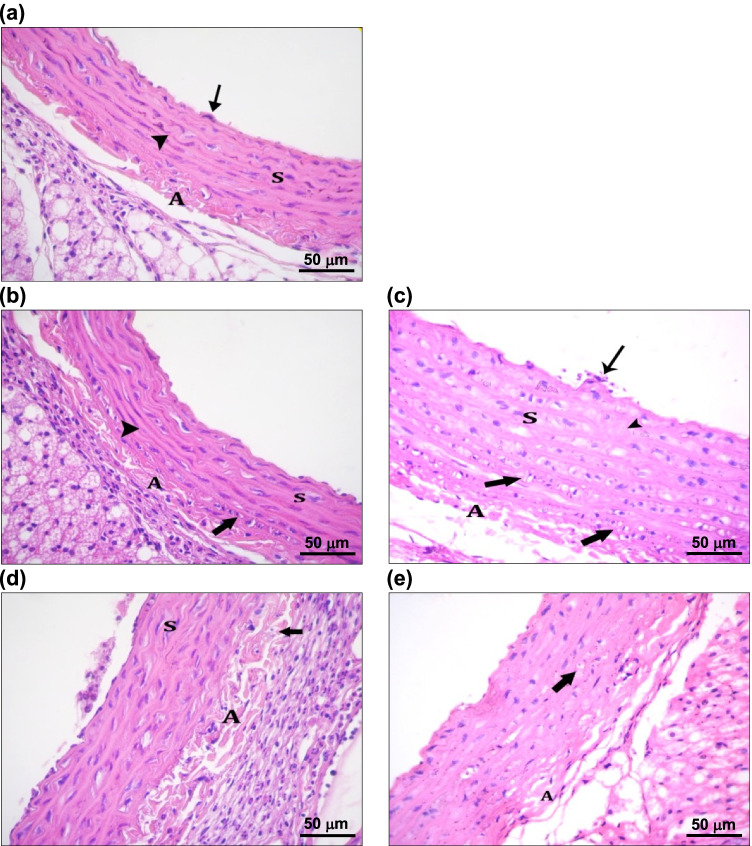
Light photomicrographs of rats’ aorta. (**a**) Young control rats showing endothelial cells (thin arrow) of tunica intima, bundles of smooth muscle cells (S) in tunica media with wavy elastic lamellae (arrow head) in-between and tunica adventitia (A) with loose connective tissue with vasa vasorum; (**b**) 5-week ovariectomized rats showing vacuolation of some smooth muscle fibers (S) with straightening of most parts of elastic lamellae (arrow head) and appearance of round to oval vacuolated cells with small pyknotic nuclei (thick arrow) known as foam cells in outer part of tunica media with inflammatory cellular infiltration in tunica adventitia (A); (**c**) 10-week young ovariectomized rats showing stickiness of blood cells to the endothelium of tunica intima (thin arrow) and vacuolation of most smooth muscle fibers (S) in tunica media with separation between elastic lamellae, which show discontinuity in some areas (arrow head) with increased number of foam cells (thick arrow) in middle and outer part of tunica media as well as tunica adventitia (A); (**d**) 1-week lipectomized rats showing vacuolation of most smooth muscle fibers (S) with the presence of foam cells (thick arrow) in outer part of tunicae media and adventitia (A) with increased inflammatory cellular infiltration; (**e**) 6-week lipectomized rats showing foam cells in all parts of tunicae media and adventitia (A) with straightening of most parts of elastic lamellae

Examination of the aorta from sham-operated rats showed normal endothelial cells of the tunica intima, bundles of smooth muscle cells in the tunica media with wavy elastic lamellae in-between, and loose connective tissue with vasa vasorum forming the tunica adventitia (Fig. 4a). The aorta of the 5-week OVX group exhibited vacuolation of some smooth muscle fibers in the outer part of tunica media with straightening of most parts of the elastic lamellae and the appearance of round to oval vacuolated cells with small pyknotic nuclei, known as foam cells. Meanwhile, the adventitia showed cellular infiltration (Fig. 4b). Ten weeks of ovariectomy resulted in blood cells sticking to the endothelium of the tunica intima accompanied by vacuolation of most smooth muscle fibers in the tunica media and separation between the elastic lamellae, which showed discontinuity in some areas. In addition, the number of foam cells was increased in the middle and outer part of the tunica media as well as in the tunica adventitia (Fig. 4c). One week after subcutaneous lipectomy, most of the smooth muscle fibers were vacuolated with foam cells in the outer part of the tunicae media and adventitia along with increased inflammatory cellular infiltration compared to only OVX rats (Fig. 4d). In the 10-week OVXL rats, foam cells were distributed throughout all the thickness of the tunicae media and adventitia, with straightening of most parts of the elastic lamellae (Fig. 4e).

### Correlation studies

**Fig. 5:**
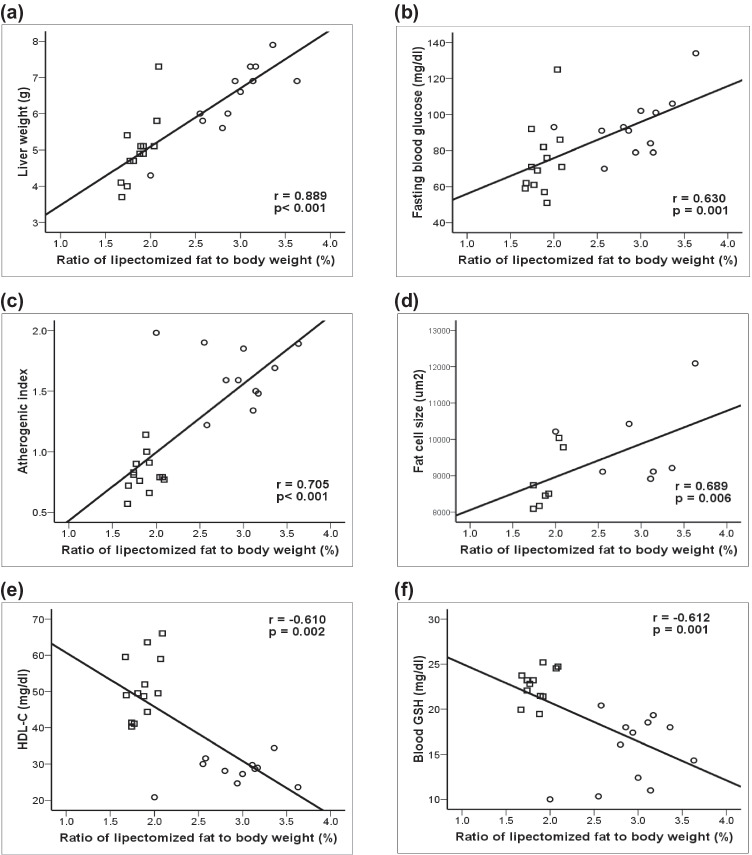
Scatter plot showing the relationship between weight of removed fat/body weight on the day of lipectomy surgery (i.e., ratio of lipectomized subcutaneous fat to body weight) in the lipectomized rat groups (○: 1-week lipectomized; □: 6-week lipectomized) and (**a**) liver weight; (**b**) fasting blood glucose; (**c**) atherogenic index; (**d**) fat cell size (i.e., average cell size in each group); (**e**) high-density lipoprotein-cholesterol (HDL-C); (**f**) blood reduced glutathione (blood GSH)

Correlation coefficient (r) between percentage ratio of lipectomized SAT/BW and other measured parameters in the lipectomized rats (both 5- and 10-week) are portrayed in Fig. (5). The ratio of lipectomized SAT showed significant positive correlation with liver weight, levels of FBG, AI, and fat cell size. On the other hand, it showed an inverse relationship with the values of HDL-C and blood GSH.

## Discussion

The present study aimed to investigate the outcomes of partial subcutaneous lipectomy in a model of ovariectomy-induced obesity in young rats. The results obtained revealed that lipectomy was followed in the long run by an increase in VAT and weight gain with increased BMI, hyperglycemia, dyslipidemia, disturbed redox status, and hepatic fatty infiltration with impaired liver functions.

Successful induction of obesity was marked by the significant increase in BW, BMI, and perirenal fat mass, and was confirmed by fat cell hypertrophy seen in the histological examination. Obese rats exhibited significant hyperglycemia and dyslipidemia (manifested by elevated plasma TC and LDL-C with increased AI), in addition to the elevated leptin level, as a result of increased adipose tissue mass. The unfavorable lipid profile was evident histologically in the liver and in aortic and perirenal fat tissue sections, all of which showed deposition of fat droplets in the functioning cells and infiltration with inflammatory cells associated with variable degrees of tissue destruction. Moreover, the decreased plasma proteins and increased liver weight of these rats are suggestive of hepatic steatosis, particularly due to the persistent elevation of ALT, which is directly linked to liver fat content [18]. Another major hazard of obesity is evidenced by a significant rise in plasma MDA level accompanied by a decrease in hepatic tissue GSH, indicating a disturbance of the pro-oxidant/antioxidant balance.

These results are consistent with those of our previous study in which ovariectomy was used to induce obesity in premenopausal rats [17]. Despite the similarity in net results and study duration, body weight and metabolic changes were more prominent in young rats than in premenopausal rats (Table 3). It was obvious that the young OVX rats displayed greater BW, BMI, and perirenal fat cell size compared to the older rats, as well as lower levels of plasma proteins and albumin. Conversely, aberrations in the lipid profile were significantly lower in young OVX rats than in their premenopausal counterparts. This could be explained by the increased BMI and fat content in these young rats and the possible role of adipose tissue as a source of estrogen. Ovariectomy has been reported to increase aromatase protein and mRNA expression in SAT, with gradual elevation of circulatory levels of estradiol [19]. Meanwhile, with age, there is a greater tendency for VAT to expand and a limited ability to increase SAT, which hinders the increase in estrogen levels [20]. In addition, although MDA levels were comparable in the two groups, both blood and hepatic GSH were higher in young rats than in premenopausal rats, confirming that the aging process is accompanied by reduced antioxidant capacity that will exacerbate obesity-induced oxidative stress [21]

**Table 3:** Body and biochemical parameters 10 weeks after ovariectomy-induced obesity in young and premenopausal rats

	Young	Premenopausal	*p*-value
Δ BW (%)	42.11 (33.33, 47.78)	23.33 (14.81, 30.36)	0.000
Δ BMI (%)	27.34 (21.15, 31.25)	23.73 (14.29, 29.51)	0.027
TG (mg/dl)	42.02 (33.61, 52.96)	58.41 (40.95, 84.96)	0.001
TC (mg/dl)	76.60 (68.10, 96.32)	91.04 (80.60, 105.97)	0.012
HDL-C (mg/dl)	49.50 (41.39, 64.91)	28.14 (20.48, 34.14)	0.000
LDL-C (mg/dl)	20.06 (13.76, 28.43)	55.13 (42.53, 66.09)	0.000
AI	0.54 (0.43, 0.78)	2.64 (1.67, 2.76)	0.000
Total proteins (g/dl)	5.94 (5.59, 6.21)	6.72 (6.09,7.76)	0.000
Albumin (g/dl)	2.99 (2.46, 3.71)	3.32 (2.96, 4.08)	0.042
Plasma MDA (nmol/ml)	122.50 (102.00, 161.00)	112.00 (93.00, 129.00)	0.062
Blood GSH (mg/dl)	24.66 (20.53, 27.13)	22.06 (19.06, 24.06)	0.032
Hepatic GSH (mg/g)	23.60 (20.67, 28.96)	7.03 (6.13, 7.85)	0.000
Fat cell size (μm^2^)	7361.39 (6133.73, 8754)	5366.69 (4099.57, 7931.80)	0.035

Values are presented as median (min, max), n = 13 for young, and n = 11 for premenopausal

Significance of differences was calculated with the Mann-Whitney U test at *p* < 0.05

*BW*, body weight; *BMI*, body mass index; *TG*, triglycerides; *TC*, total cholesterol; *HDL-C*, high-density lipoprotein-cholesterol; *LDL-C*, low-density lipoprotein-cholesterol; *AI*, atherogenic index; *MDA*, malondialdehyde; *GSH*, reduced glutathione

### Effects of subcutaneous lipectomy

One week following partial subcutaneous lipectomy, the % change in BW and BMI was significantly decreased. However, this reduction was associated with significant increase in perirenal fat cell size compared to both sham-operated and OVX rats (Fig. 2a), pointing to the triggering of body compensatory mechanisms. This compensation was evident 6 weeks after lipectomy, where the rats gained weight and their BMI became statistically indistinguishable from that of the corresponding only OVX group. It was also accompanied by both an increase in perirenal fat mass and fat cell size. Several studies have reported a compensatory increase in VAT following the removal of SAT in humans [22, 23]. It was postulated that when body fat is surgically removed, it will be recovered by compensatory expansion at intact depots rather than regrowth of the fat mass in aspirated depots, with a predominant hypertrophy of the retroperitoneal pad of fat [24, 25]. This might explain the significant increase in perirenal fat mass and fat cell size observed in the present study. It also matched the significant positive correlation encountered between the percentage of the excised SAT and fat cell size of remaining VAT (Fig. 5d).

Lipectomy resulted in a significant drop in plasma leptin reaching the level of that in sham-operated rats. Leptin levels have been documented to be correlated with the amount of body fat mass, and it is known to be preferentially secreted from SAT compared with VAT [26]. Thus, the decrease in leptin level in the present study corresponds to the removal of SAT, with a subsequent decline in its secretion. Interestingly, leptin levels failed to increase 6 weeks following lipectomy despite the increase in BMI and regeneration of body fat. This may be due to failure of regrowth of SAT with regeneration of adipose tissue occurring in other areas, as manifested by increased fat cell size in perirenal fat. Adiponectin is another adipocyte-derived hormone whose level was comparable in all the studied groups whether before or after lipectomy. Reports about changes in adiponectin level after lipectomy are controversial, with some studies reporting no change [27] and others reporting its increase [28], or decrease [29].

Ovariectomized lipectomized rats exhibited significant hyperglycemia, which could be explained by the increased proportion of VAT relative to SAT and, hence, increased rate of lipolysis, providing a continuous substrate for gluconeogenesis [8, 30]. The removal of SAT was reported to enhance ectopic fat deposition in liver and skeletal muscles, which is associated with insulin resistance [31]. This was confirmed by the presence of significant positive correlation between the percentage of the excised SAT and levels of FBG (Fig. 5b). Moreover, the lower leptin levels in lipectomized rats could indicate another mechanism to explain the increased FBG. Leptin was documented to improve glucose tolerance via reduction of fat deposited ectopically [32], exerting insulin-like effects on skeletal muscles [33] and inhibiting glucagon secretion from pancreatic cells [34]. However, this concept is not entirely valid as, despite the high leptin levels following ovariectomy, it did not correct the hyperglycemia or protect against the deterioration in lipid profile.

Subcutaneous lipectomy seems to cause further worsening of the atherogenic lipid profile encountered in the only OVX rats. The TC, LDL-C, and AI were all still elevated in addition to the significant rise in plasma TG and reduction of HDL-C levels. It was also found that the ratio of the excised SAT was positively correlated with the calculated AI and inversely correlated with the plasma levels of HDL-C (Fig. 5c,e). This could possibly be attributed to lack of the protective effect of SAT, which is dubbed the “metabolic sink” for dietary fat [35]. In the current study, the acute removal of subcutaneous fat and the disturbed subcutaneous/visceral fat ratio resulted in a rebound increase of visceral fat, manifested by accumulation of fat in the perirenal adipocytes. The larger perirenal adipocytes observed in lipectomized rats suggest another explanation for the worsened lipid profile. It has been postulated that when adipocytes enlarge, the activity of lipoprotein lipase increases in parallel, which further increases fatty acid delivery to the circulation [36]. Adipocyte hypertrophy is also linked to impaired adipose tissue function, including high responses to lipolytic agonists and lower diacyl glycerol synthase activity [37].

Six weeks following lipectomy, the atherogenic lipid profile and hyperglycemia still persisted, with the exception of TG and HDL-C levels which were significantly improved. The significant reduction of HDL-C after 1 week of lipectomy could be explained by the observed upregulation of enzymes involved in HDL-C catabolism following lipectomy [38]. Interestingly, Catapano et al. [39] reported that during infections or acute medical conditions, HDL-C levels decrease very rapidly and the particles undergo profound changes in their composition and function. In the present study, we might consider the lipectomy surgery as having been an acute medical condition, constituting a second surgical intervention within a month. Meanwhile, the significant decrease in TG level compared to its value in 1-week lipectomized rats could be attributed to the buffering of excess free fatty acids in plasma by the compensatory increase in adipose tissue and total body fat in this group, with subsequent lowering of circulating TG level [40].

The impact of lipectomy on the liver is marked by increased liver weight and reduction in albumin level, pointing to deterioration of hepatic function. The presence of this effect was supported by the fact that reduction of a significant amount of SAT was associated with a trend toward increased fatty infiltration of the liver and enhancement of ectopic lipid deposition due to removal of the natural store [31, 41]. The effect was also confirmed by the significant positive correlation detected between the ratio of excised SAT and hepatic weight in the OVXL groups (Fig. 5a). The drop in the level of hepatic enzymes in OVXL rats, though an abnormal histological picture, probably indicates severe liver affection resulting in fewer liver cells that can leak these enzymes [42].

Subcutaneous lipectomy resulted in more disturbance of redox status, manifested by the continuous increase in plasma MDA level and reduction in both blood and hepatic tissue GSH. The increased oxidative stress with lipectomy could be, in part, explained by the removal of SAT, which enhances ectopic fat deposition and is known to be associated with inflammation [31]. The link between lipectomy and oxidative stress was demonstrated by a significant negative correlation between the ratio of excised SAT and values of blood GSH in the OVXL groups (Fig. 5f).

### Subcutaneous lipectomy in young versus premenopause rats

The present data extend and confirm our previous research on the impact of subcutaneous lipectomy in obese premenopausal rats [17]. The results demonstrated that lipectomy surgery, whether in premenopausal or in young rats, has unfavorable outcomes, including fasting hyperglycemia, disturbed lipid profile associated with atherosclerotic changes in the wall of the abdominal aorta, and impaired liver functions coupled with hepatic cell vacuolation and inflammatory cell infiltration. Furthermore, there was an increase in the level of MDA with decreased antioxidant capacities and an increase in the size of visceral fat cells. The increased liability for atherosclerotic changes, together with enhanced ectopic fat deposition in the liver and the wall of the aorta could be related to the drop of leptin level observed in these rats.

**Fig. 6:**
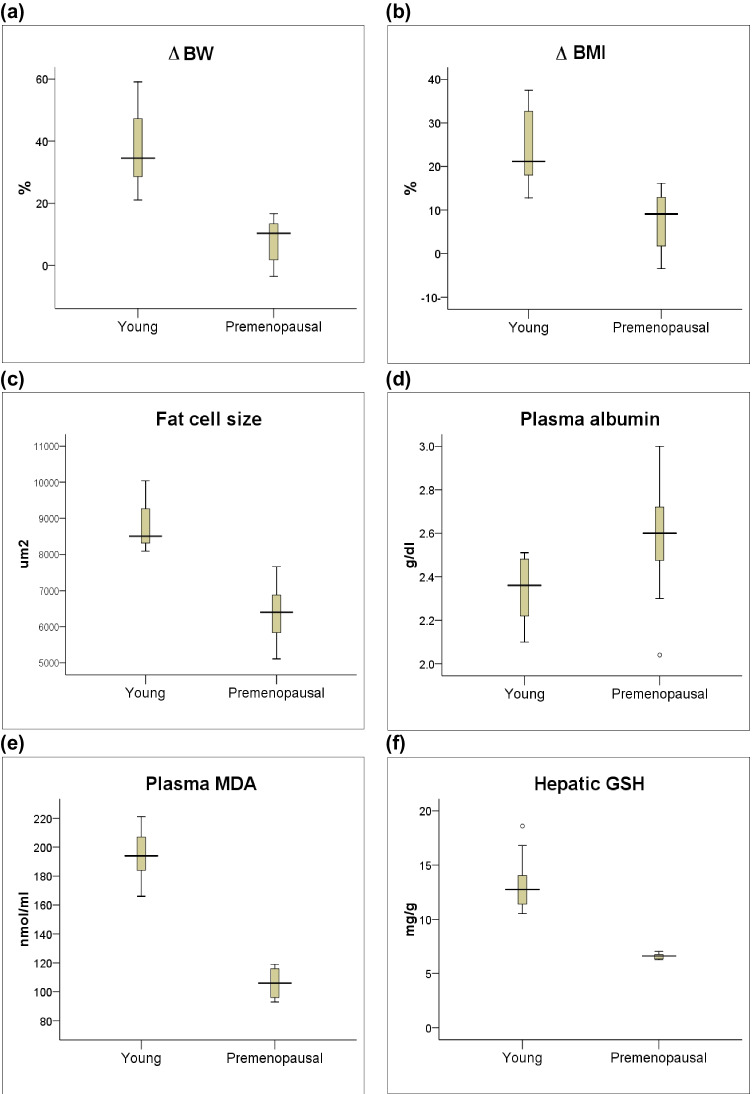
Body responses 6 weeks after subcutaneous adipose tissue lipectomy in young and premenopausal rats. Values are presented as median (min, max), n = 13 for young, and n = 11 for premenopausal. Significance of differences was calculated with the Mann-Whitney U test at *p* < 0.05. BW: body weight; BMI: body mass index; MDA: malondialdehyde; GSH: reduced glutathione

Furthermore, subcutaneous lipectomy appears not to be effective in combating ovariectomy-induced obesity in young rats, the unfavorable effects on both metabolic and hepatic functions seeming to be more pronounced at young ages. Compared with premenopausal rats, young rats appear to have compensated for the removed fat tissue as evidenced by the percentage increase in BW [34.52 (21.05, 59.09) vs. 10.34 (−3.57, 16.67), *p* < 0.001, Fig. 6a] and BMI [21.15 (12.77, 37.50) vs. 9.09 (−3.45, 16.13), *p* < 0.001, Fig. 6b], as well as increased perirenal fat cell size [8504.87 (8089.34, 10037.91) vs. 6402.19 (5110.61, 7654.64), *p* = 0.002, Fig. 6c]. In premenopausal rats, the limited ability of adipose tissue to regenerate could be correlated to the decline in preadipocyte replication and adipogenesis and, moreover, in adipocyte ability to synthesize and store neutral fat with aging [43]. This limited capacity hindered the compensatory increase in body fat content and body weight of aged rats following lipectomy.

In young rats, subcutaneous lipectomy was followed by significant reduction in plasma albumin level compared to premenopausal rats [2.36 (2.10, 2.51) vs. 2.60 (2.04, 3.00), *p* = 0.009, Fig. 6d]. Albumin is the most important plasma protein synthesized by the liver and is believed to be a useful indicator of hepatic function; therefore, a low level provides evidence of deterioration [44]. Elevated levels of plasma MDA [194.00 (166.00, 221.00) vs. 106.00 (93.00, 119.00), *p* < 0.001, Fig. 6e] and hepatic tissue GSH [12.75 (10.55, 18.61) vs. 6.61 (6.26, 7.03), *p* < 0.001, Fig. 6f] in young rats compared to premenopausal ones indicate high redox status after lipectomy. The low levels of hepatic GSH in premenopausal rats could be explained by the aging process. It has been reported that aging is characterized by increased intracellular oxidative stress due to the progressive decrease in intracellular scavenging of reactive oxygen species [45].

In fact, studies involving surgical manipulation of adipose tissue by removal or partial lipectomy have inconsistent outcomes. There is some debate as to whether lipectomy itself is ineffective, or whether there is an underlying problem of energy imbalance that has not been corrected. Despite the variable results obtained after lipectomy, most experimental studies demonstrated body fat compensation and weight recovery, suggesting that biological feedback mechanisms act to resist long-term changes in body weight/fat [46]. This is because the body aims to maintain an energy balance that requires a complex integration of energy stores, energy expenditure, and energy intake. However, in the long term, it is now clear that surgically eliminating fat stores without correcting the energy balance simply results in regrowth of fat mass either at the excision site or (more commonly) in other depots.

In this context, partial lipectomy was found to increase lipogenesis and adipocyte differentiation in non-excised depots in a model of obesity induced by monosodium glutamate (MSG) treatment [25]. Recently, though using the same model of obesity (i.e., MSG treatment), lipectomy in obese animals resulted in significantly higher visceral fat accumulation in female than in male rats, pointing to a gender-dependent difference [47]. In another study, the effect of partial lipectomy on high fat diet-induced obesity in rats was evaluated. The results showed that although there was no significant difference in food intake among all groups, the lipectomized animals had higher weight and greater fat accumulation in the liver than the control group [48]. These data indicate that lipectomy tends to enhance the anabolic pathways but leaves the catabolic pathways unaffected. Meanwhile, Habitante et al. [49] have shown that exercise training after partial removal of fat pads modifies adipose tissue metabolism, impairs adipose tissue regeneration, and reduces body adiposity.

Although lipectomy models offer some insights into how lipid (energy) stores and body composition are regulated, the interactive effects of other factors have not been well defined. For a better understanding, more empirical studies in different contexts such as genetic factors, different diets, exercise, and environmental conditions (e.g., photoperiod and temperature) are needed. The novelty of the present study, in the absence of adequate number of research in female animals, is the reporting of significant changes in metabolic parameters in lipectomized young obese female rats compared to non-lipectomized ones. It is the effect of the sudden shortage of energy storage on the metabolic profile that leads to metabolic reprogramming in the liver, which is the most important organ responsible for regulating energy metabolism.

## Conclusion

Subcutaneous lipectomy appears to be ineffective in combating obesity at young ages. Despite the rapid and significant loss of BW immediately after lipectomy, this outcome was found to be temporary and was followed by compensatory expansion of visceral adipose tissue in multiple areas as well as in different non-adipose tissue organs. This ectopic fat deposition is thought to be responsible for the deleterious metabolic effects of lipectomy. It is thus evident that more attention must be paid to the removal of this easily accessible subcutaneous fat while bearing in mind the unfavorable outcomes of lipectomy.

## References

[1] Ganong WF (2019) Cellular and molecular basis for medical physiology. In: Barrett KE, Barman SM, Boitano S, Brooks HL (eds) Ganong’s Review of Medical Physiology, 26th edn. Lange Medical books, The Mc Graw Hill Companies, pp 27

[2] Kelesidis T, Kelesidis I, Chou S, Mantzoros CS (2010). Narrative review: the role of leptin in human physiology: emerging clinical applications. Ann Intern Med.

[3] Turer AT, Scherer PE (2012). Adiponectin: mechanistic insights and clinical implications. Diabetologia.

[4] Qi Y, Nie Z, Lee YS, Singhal NS, Scherer PE, Lazar MA (2006). Loss of resistin improves glucose homeostasis in leptin deficiency. Diabetes.

[5] Kovacs P, Geyer M, Berndt J, Klötin N, Graha TE, Böttcher Y (2007). Effects of genetic variation in the human retinol binding protein-4 gene (RBP4) on insulin resistance and fat depot-specific mRNA expression. Diabetes.

[6] Poulos SP, Hausman DB, Hausmanm GJ (2010). The development and endocrine functions of adipose tissue. Mol Cell Endocrinol.

[7] Smith SR, Lovejoy JC, Greenway F, Ryan D, deJonge L, de la Bretonne J (2001). Contributions of total body fat, abdominal subcutaneous adipose tissue compartments, and visceral adipose tissue to the metabolic complications of obesity. Metabolism.

[8] Gil A, Olza J, Gil-Campos M, Gomez-Llorente C, Aguilera CM (2011). Is adipose tissue metabolically different at different sites?. Int J Pediatr Obes.

[9] Sacks HS, Fain JN (2007). Human epicardial adipose tissue: a review. Am Heart J.

[10] Lee MJ, Wu Y, Fried SK (2013). Adipose tissue heterogeneity: implication of depot differences in adipose tissue for obesity complications. Mol Asp Med.

[11] Pitombo C, Araújo EP, De Souza CT, Pareja JC, Geloneze B, Velloso LA (2006). Amelioration of diet-induced diabetes mellitus by removal of visceral fat. J Endocrinol.

[12] Foster MT, Softic S, Caldwell J, Kohli R, deKloet AD, Seeley RJ (2013). Subcutaneous adipose tissue transplantation in diet-induced obese mice attenuates metabolic dysregulation while removal exacerbates it. Phys Rep.

[13] Foster MT, Shi H, Seeley RJ, Woods SC (2011). Removal of intra-abdominal visceral adipose tissue improves glucose tolerance in rats: role of hepatic triglyceride storage. Physiol Behav.

[14] Avci P, Nyame TT, Gupta GK, Sadasivam M, Hamblin MR (2013). Low-level laser therapy for fat layer reduction: a comprehensive review. Lasers Surg Med.

[15] Pegington M, French DP, Harvie MN (2020). Why young women gain weight: a narrative review of influencing factors and possible solutions. Obes Rev.

[16] Oguoma VM, Coffee NT, Alsharrah S, Abu-Farha M, Al-Refaei FH, Al-Mulla F (2021). Prevalence of overweight and obesity, and associations with socio-demographic factors in Kuwait. BMC Public Health.

[17] El-Kafoury BMA, Bahgat NM, Abdel-Hady EA, Abd El Samad AA, Shawky MK, Mohamed FA (2019). Impaired metabolic and hepatic functions following subcutaneous lipectomy in adult obese rats. Exp Physiol.

[18] Elizondo-Montemayor L, Ugalde-Casas PA, Lam-Franco L, Bustamante-Careaga H, Serrano-González M, Gutiérrez NG (2014). Association of ALT and the metabolic syndrome among Mexican children. Obes Res Clin Pract.

[19] Zhao H, Tian Z, Hao J, Chen B (2005). Extragonadal aromatization increases with time after ovariectomy in rats. Reprod Biol Endocrinol.

[20] Cartwright MJ, Tchkonia T, Kirkland JL (2007). Aging in adipocytes: potential impact of inherent, depot-specific mechanisms. Exp Gerontol.

[21] Salmon AB (2016). Beyond diabetes: does obesity-induced oxidative stress drive the aging process?. Antioxidants.

[22] Hernandez TL, Kittelson JM, Law CK, Ketch LL, Stob NR, Lindstrom RC (2011). Fat redistribution following suction lipectomy: defense of body fat and patterns of restoration. Obesity (Silver Spring).

[23] Benatti F, Solis M, Artioli G, Montag E, Painelli V, Saito F (2012). Liposuction induces a compensatory increase of visceral fat which is effectively counteracted by physical activity: a randomized trial. J Clin Endocrinol Metab.

[24] Hausman DB, Lu J, Ryan DH, Flatt WP, Harris RB (2004). Compensatory growth of adipose tissue after partial lipectomy: involvement of serum factors. Exp Biol Med (Maywood).

[25] Bueno AA, Oyama LM, Estadella D, Habitante CA, Bernardes BS, Ribeiro EB (2005). Lipid metabolism of monosodium glutamate obese rats after partial removal of adipose tissue. Physiol Res.

[26] Koerner A, Kratzsch J, Kiess W (2005). Adipocytokines: leptin--the classical, resistin--the controversical, adiponectin--the promising, and more to come. Best Pract Res Clin Endocrinol Metab.

[27] Ybarra J, Blanco-Vaca F, Fernández S, Castellví A, Bonet R, Palomer X (2008). The effects of liposuction removal of subcutaneous abdominal fat on lipid metabolism are independent of insulin sensitivity in normal-overweight individuals. Obes Surg.

[28] Rizzo MR, Paolisso G, Grella R, Barbieri M, Grella E, Ragno E (2005). Is dermolipectomy effective in improving insulin action and lowering inflammatory markers in obese women?. Clin Endocrinol.

[29] Busetto L, Bassetto F, Zocchi M, Zuliani F, Nolli ML, Pigozzo S (2008). The effects of the surgical removal of subcutaneous adipose tissue on energy expenditure and adipocytokine concentrations in obese women. Nutr Metab Cardiovasc Dis.

[30] Boden G (1996). Fatty acids and insulin resistance. Diabetes Care.

[31] Kim JY, van de Wall E, Laplante M, Azzara A, Trujillo ME, Hofmann SM (2007). Obesity-associated improvements in metabolic profile through expansion of adipose tissue. J Clin Invest.

[32] Brennan AM, Mantzoros CS (2006). Drug insight: the role of leptin in human physiology and pathophysiology--emerging clinical applications. Nat Clin Pract Endocrinol Metab.

[33] Mantzoros CS, Magkos F, Brinkoetter M, Sienkiewicz E, Dardeno TA, Kim SY (2011). Leptin in human physiology and pathophysiology. Am J Physiol Endocrinol Metab.

[34] Tudurí E, Marroquí L, Soriano S, Ropero AB, Batista TM, Piquer S (2009). Inhibitory effects of leptin on pancreatic alpha-cell function. Diabetes.

[35] Porter SA, Massaro JM, Hoffmann U, Vasan RS, O'Donnel CJ, Fox CS (2009). Abdominal subcutaneous adipose tissue: a protective fat depot?. Diabetes Care.

[36] Wajchenberg BL (2000). Subcutaneous and visceral adipose tissue: their relation to the metabolic syndrome. Endocr Rev.

[37] Michaud A, Boulet MM, Veilleux A, Noël S, Paris G, Tchernof A (2014). Abdominal subcutaneous and omental adipocyte morphology and its relation to gene expression, lipolysis and adipocytokine levels in women. Metabolism.

[38] Syvänne M, Taskinen MR (1997). Lipids and lipoproteins as coronary risk factors in non-insulin-dependent diabetes mellitus. Lancet.

[39] Catapano AL, Pirillo A, Bonacina F, Norata GD (2014). HDL in innate and adaptive immunity. Cardiovasc Res.

[40] Frayn KN (2002). Adipose tissue as a buffer for daily lipid flux. Diabetologia.

[41] Ibrahim MM (2010). Subcutaneous and visceral adipose tissue: structural and functional differences. Obes Rev.

[42] Porter RS, Kaplan JL, Sharp M, Dohme (2011) Hepatic and biliary disorders. In: Published by Merck Sharp & Dohme Corp., a subsidiary of Merck & CO., INC (ed) In: The Merck Manual of Diagnosis and Therapy, Whitehouse Station, pp 203–281

[43] Tchkonia T, Morbeck DE, Von Zglinicki T, Van Deursen J, Lustgarten J, Scrable H (2010). Fat tissue, aging, and cellular senescence. Aging Cell.

[44] Thapa BR, Walia A (2007). Liver function tests and their interpretation. Indian J Pediatr.

[45] Minelli A, Bellezza I, Conte C, Culig Z (2009). Oxidative stress-related aging: a role for prostate cancer?. Biochim Biophys Acta.

[46] Murillo AL, Kaiser KA, Smith DL, Peterson CM, Affuso O, Tiwari HK, Allison DB (2019). A systematic scoping review of surgically manipulated adipose tissue and the regulation of energetics and body fat in animals. Obesity (Silver Spring).

[47] Pimenta FS, Tose H, Waichert É, da Cunha MRH, Campos FV, Vasquez EC (2019). Lipectomy associated to obesity produces greater fat accumulation in the visceral white adipose tissue of female compared to male rats. Lipids Health Dis.

[48] Ling BL, Chiu CT, Lu HC, Lin JJ, Kuo CY, Chou FP (2014). Short and long-term impact of lipectomy on expression profile of hepatic anabolic genes in rats: a high fat and high cholesterol diet-induced obese model. PLoS One.

[49] Habitante CA, Oyama LM, Bueno AA, Ribeiro EB, Estadella D, Dâmaso AR (2010). Exercise training in rats impairs the replenishment of white adipose tissue after partial lipectomy. Eur J Appl Physiol.

